# A Chinese patient with 11β-hydroxylase deficiency due to novel compound heterozygous mutation in *CYP11B1* gene: a case report

**DOI:** 10.1186/s12902-018-0295-6

**Published:** 2018-09-21

**Authors:** Xianxian Yuan, Lin Lu, Shi Chen, Jun Jiang, Xiangqing Wang, Zhihui Liu, Huijuan Zhu, Hui Pan, Zhaolin Lu

**Affiliations:** 1 Department of Endocrinology, Peking Union Medical College Hospital, Chinese Academy of Medical Science and Peking Union Medical College, Key Laboratory of Endocrinology of National Health Commission of the People’s Republic of China, No. 1 Shuaifuyuan, Dongcheng District, Beijing, 100730 China; 2 The Key Laboratory of Genome Sciences and Information, Beijing Institute of Genomics, Chinese Academy of Sciences, Beijing, 100101 China

**Keywords:** 11β-hydroxylase deficiency, *CYP11B1* gene mutation, Iatrogenic Cushing’s syndrome

## Abstract

**Background:**

Congenital adrenal hyperplasia (CAH) resulting from steroid 11β-hydroxylase deficiency (11β-OHD) is caused by mutations in the *CYP11B1* gene. It is the second major form of CAH associated with hypertension and hypopotassemia. The aim of this study was to provide a genetic analysis of 11β-OHD in a Chinese family.

**Case presentation:**

A 19-year-old Chinese man was clinically diagnosed with 11β-OHD. His initial clinical manifestations included precocious puberty, hyperpigmentation, hypertension, and hypopotassemia. The patient had taken an overdose of dexamethasone (0.75 mg/d) for more than 10 years before finally developing iatrogenic Cushing’s syndrome. Our aim was to perform a molecular diagnosis of his family. Mutations in the *CYP11B1* gene of the patient and his parents were examined using polymerase chain reaction (PCR) resequencing. Additionally, to predict the possible effects of novel mutations on the structure and function of 11β-hydroxylase, these mutations were analyzed by MutationTaster software. Two novel pathogenic mutations were found in the *CYP11B1* gene: a heterozygous in-frame insertion deletion mutation c.1440_1447delinsTAAAAG in exon 9 inherited from the father and a heterozygous mutation c.1094_1120delTGCGTGCGGCCCTCAAGGAGACCTTGC (p.364_372del) in exon 6 inherited from the mother.

**Conclusions:**

A clear genetic diagnosis can be made by analyzing the functional and structural consequences of *CYP11B1* gene mutations that lead to 11β-OHD. Because the dosage of glucocorticoid should be adjusted to minimize the risk of iatrogenic Cushing’s syndrome, clinical follow-up should be conducted with these patients.

**Electronic supplementary material:**

The online version of this article (10.1186/s12902-018-0295-6) contains supplementary material, which is available to authorized users.

## Background

Congenital adrenal hyperplasia (CAH) is an autosomal recessive disorder caused by the loss or severe decrease in activity in one of the steroidogenic enzymes involved in cortisol biosynthesis. The majority of cases are caused by 21-hydroxylase deficiency (21-OHD) [1–3], followed by 11β-hydroxylase deficiency (11β-OHD) in Jews of Moroccan origin, people of Turkish descent, and Han Chinese. In the general Caucasian population, it has a prevalence of 5–8% and occurs in approximately 1:100,000 to 1:200,000 of live births [4–7].

The 11β-hydroxylase converts 11-deoxycortisol and 11-deoxycorticosterone (DOC) to cortisol and corticosterone, respectively, and it is regulated by the adrenocorticotropic hormone (ACTH) secreted by the pituitary gland. A deficit of 11β-hydroxylase, however, causes decreased production of cortisol, accumulation of steroid precursors and an overproduction of androgens. Therefore, the classical form of 11β-OHD is characterized by genital ambiguity in female patients [4] and precocious puberty, rapid somatic growth and bone age acceleration in both genders as a result of hyperandrogenemia. Hypertension occurs in approximately two-thirds of these patients because of the accumulation of steroid precursors, primarily deoxycorticosterone. In contrast, this symptom is not seen in patients with 21-OHD.

The 11β-OHD is caused by the mutation of the 11β-hydroxylase gene (*CYP11B1*), which is located on chromosome 8q21. This gene contains 9 exons, approximately 40 kilobases apart from the highly homologous aldosterone synthase gene (*CYP11B2*) [8, 9]. Herein, we first report the observation of classic features with two novel mutations genetically confirmed for 11β-OHD in a Chinese family.

## Case presentation

**Fig. 1:**
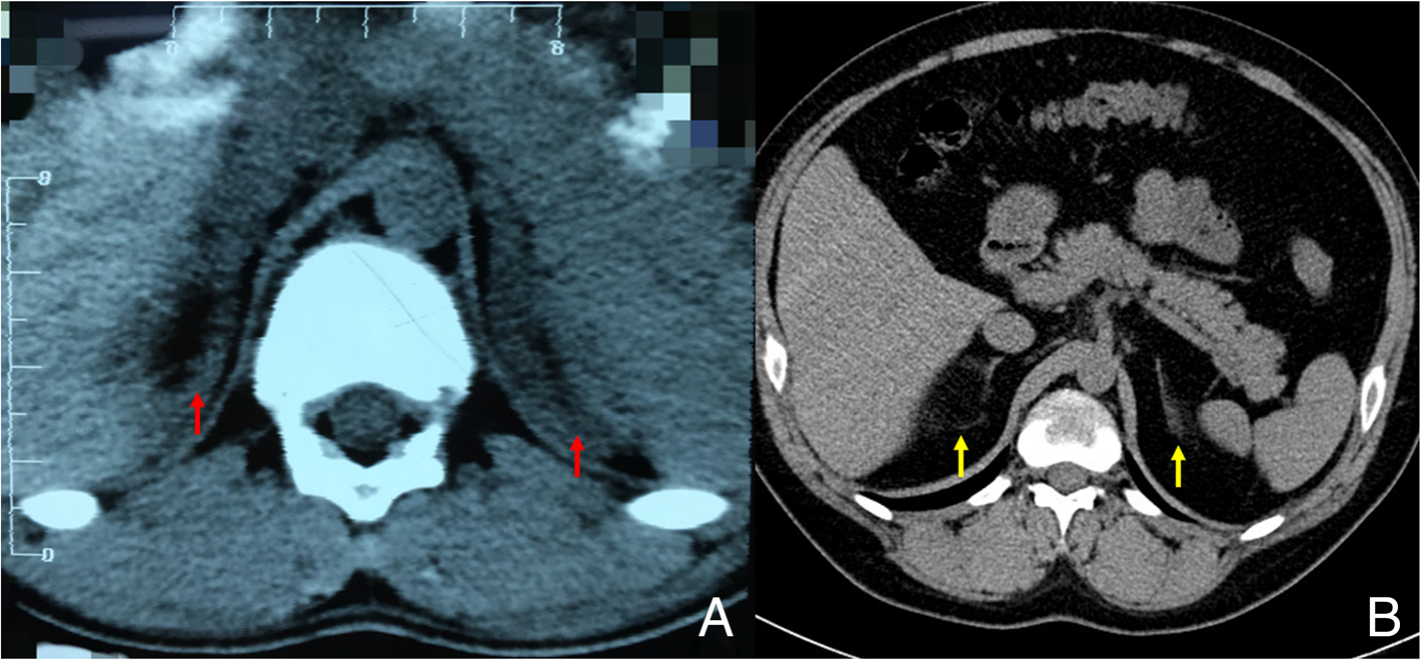
Adrenal computed tomographic scan. **a** 13 years ago (age: 7 years, 3 months), before treatment, the scan revealed bilateral adrenal hyperplasia (red arrow). **b** On admission (age: 19 years), after treatment with dexamethasone, the scan showed bilateral adrenal atrophy (yellow arrow)

The patient was born after a full-term delivery by natural labor, and his parents had a non-consanguineous marriage. A large phallus and skin hyperpigmentation were observed at birth. He presented with deep voice and accelerated growth rate at the age of 12 months. At 6.5 years, the appearance of pubic hair was reported. He was admitted to a pediatric hospital at the age of 7.3 years. His height was 144.5 cm (+ 3.6 SDS, according to the 2009 height standardized growth chart for Chinese children and adolescents aged 2 to 18 years old). He presented with more dark skin, a larger phallus, advanced bone age, and high blood pressure (160/100 mmHg). A laboratory investigation showed the following results: potassium 3.06 mmol/L; sodium 142.1 mmol/L; testosterone more than 750 ng/dL; follicle stimulating hormone (FSH) 27.2 mIU/ml; luteinizing hormone (LH) 4.2 mIU/ml; morning serum cortisol 1.1 μg/dl; and adrenocorticotropic hormone (ACTH) 673 pg/ml (Table 1). A computed tomography scan showed bilateral adrenal enlargement, as shown in Fig. 1a. He was diagnosed with congenital adrenal hyperplasia resulting from 11β-OHD and began the dexamethasone treatment. After taking dexamethasone, the patient achieved normal sodium and potassium and decreased blood pressure (130/80 mmHg).

**Table 1:** Biochemical and hormonal findings of the patient before and after treatment with glucocorticoid

Biochemical and hormonal findings	13 years ago	On admission	1 month later	4 months later	Normal values
Treatment	Before treatment	Dexamethasone (0.75 mg/d)	Prednisone (7.5 mg/d)	Prednisone (7.5 mg/d)	
Na^+^ (mmol/L)	142.1	137	138	135	135–145
K^+^ (mmol/L)	3.06	5.0	4.0	4.7	3.5–5.5
Cl^−^ (mmol/L)	–	102	103	100	96–111
P (ng/ml)	–	0.16	0.21	0.37	0.10–0.84
17-OHP (ng/ml)	–	1.66	1.45	2.32	<0.7–2.5
T (ng/ml)	>750 ng/dl^a^	2.33	2.51	3.13	1.75–7.81
DHEA-S (μg/dl)	–	26.9	25.2	–	24–537
E_2_ (pg/ml)	–	<5	31	35.0	<47
LH (IU/L)	4.2	5.08	5.02	3.68	1.24–8.62
FSH (IU/L)	27.2	8.85	5.76	4.73	1.27–19.26
Cortisol at 8 AM (μg/dl)	1.1	–	1.24	–	5–25
ACTH at 8 AM (pg/ml)	673	<5	24.8	12.5	0–46
PRA upright (ng/ml/h)	0.005	8.27	2.26	–	0.93–6.56
AT-II upright (pg/ml)	82.14	112.18	94.09	–	25.3–145.3
Aldo upright (ng/dl)	6.16	13.24	6.73	–	6.5–29.6
UA (μmol/L)	–	706	730	620	210–416
TC (mmol/L)	–	6.21	5.42	6.04	2.85–5.70
TG (mmol/L)	–	2.17	1.25	1.20	0.45–1.70
HDL-C (mmol/L)	–	1.86	1.29	1.40	0.93–1.81
LDL-C (mmol/L)	–	3.67	3.58	3.79	<3.37

Endnote: ^a^*T* = 750 ng/dl = 7.5 ng/ml (normal value 0–45 ng/dl)

**Fig. 2:**
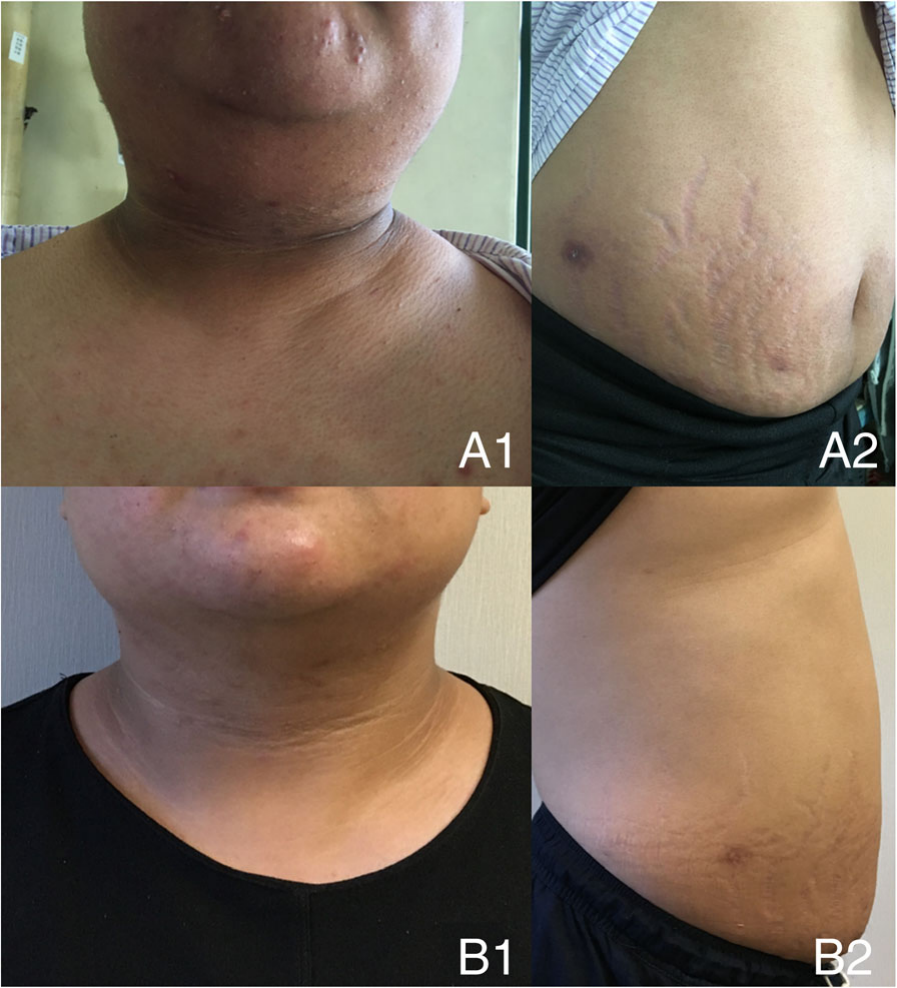
General appearance of the patient. (A) At the neck, clavicle fat pad and acanthosis nigricans was found. (B) On his lower trunk, central obesity and striae were noted. (1) Before changing medication from dexamethasone to prednisone. (2) Four months after changing medication from dexamethasone to prednisone

The patient took dexamethasone 0.75 mg/d for more than 10 years without follow-up or dose titration, and gradually developed symptoms of weight gain, round face, ecchymoses, striae, acne, hyperuricemia, and recurrent tinea corporis. Additionally, his blood pressure rose to 150/100 mmHg. He reached his final adult height at 8 years old (148 cm, + 3.2 SDS). The patient was referred to our hospital with the complaint of short stature when he was 19 years old (148 cm, − 4.0 SDS). At this visit, he presented with blood pressure of 160/120 mmHg, a body mass index (BMI) of 36.5 kg/m^2^, and a waist circumference of 110 cm, with the appearance of typical Cushing’s syndrome with neck acanthosis nigricans (Fig. 2). The laboratory test results showed the following (Table 1): ACTH less than 5 pg/ml; cortisol 1.24 μg/dl; testosterone 2.33 ng/ml; progesterone 0.16 ng/ml; 17α-hydroxyprogesterone 1.66 ng/ml; dehydro-epiandrosterone sulfate (DHEA-S) 26.9 μg/ml; potassium 5.0 mmol/L and sodium 137 mmol/L. A computed tomography scan revealed bilateral adrenal atrophy (Fig. 1B). He was diagnosed with hyperinsulinemia, hyperuricemia, hyperlipidemia, and fatty liver. The patient was diagnosed with iatrogenic Cushing’s syndrome. Due to significantly inhibition ACTH, elevated plasma renin activity, and bilateral adrenal atrophy, we speculated that the patient was deficient in mineralocorticoids. He stopped taking dexamethasone and began prednisone (7.5 mg/d). The patient was prescribed nifedipine controlled-release tablets (60 mg/d) and metoprolol succinate sustained-release tablets (47.5 mg/d), and his blood pressure was controlled at 138–148/90–106 mmHg. Four months after switching from dexamethasone to prednisone, the patient came to our clinic for follow-up and had decreased body weight (6 kg), normal blood pressure (120/70 mmHg), and normal serum potassium levels.

Written informed consent was obtained from the patient and his parents before the genetic investigation. *CYP11B1* (NM_000497) gene was analyzed by direct sequencing using genomic DNA extracted from leucocytes of peripheral blood by EZNA Blood DNA Midi Kit (Atlanta, GA, US). Briefly, PCR was performed in a 20 μL reaction volume containing 50 ng of genomic DNA, 10 μL of 2 × GC PCR buffer, 0.1 μM of each dNTP, 0.1 μM of each primer, and 1.25 units of rTaq polymerase (Takara, Shiga, Japan) in a thermocycler (ABI9700, US). For all of the amplicons, the genomic DNA was denatured at 94 °C for 10 min, followed by 35 cycles of denaturation at 94 °C for 30 s, 60 °C for 30 s, and 72 °C for 1 min 40 s. The final extension was at 72 °C for 10 min. To prevent amplification of highly homologous *CYP11B2* sequences, the *CYP11B1* gene was amplified in four fragments using four unique primer pairs (Additional file 1: Table S1: Primers used for PCR assay of *CYP11B1* gene) [10, 11]. Amplified products were detected by agarose gel electrophoresis and sequenced using an ABI3730 DNA Analyzer (Applied Biosystems). Each individual was identified according to the sequencing results using SeqMan software. To predict the functional effects of novel mutations, the sequence alterations were assessed using in silico prediction algorithms in MutationTaster (an online program at http://www.mutationtaster.org, which automatically provides the probability for a variation to be either a pathogenic mutation or a benign polymorphism.

**Fig. 3:**
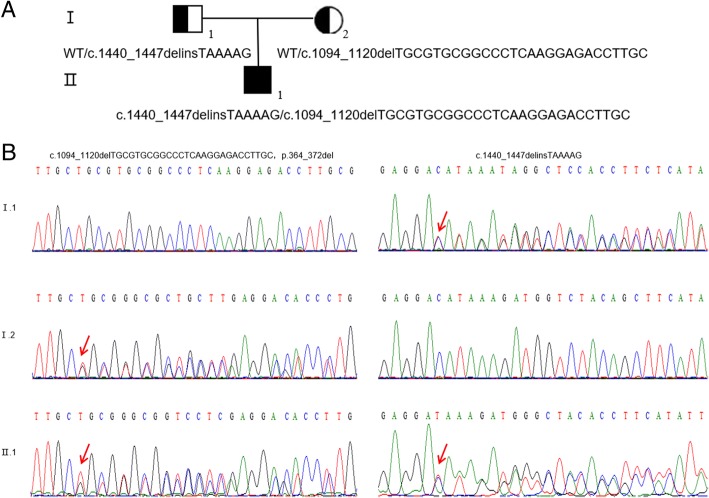
Mutation analysis by direct DNA sequencing. **a** Pedigree illustrating the segregation of the mutant alleles to the index patient (II.1). **b** The left panel shows the deletion at c.1094_1120delTGCGTGCGGCCCTCAAGGAGACCTTGC, which results in the deletion of nine amino acids at position 364_372 (p.364_372del). The mother (I.2) and the patient (II.1) are heterozygous for c.1094_1120delTGCGTGCGGCCCTCAAGGAGACCTTGC mutation, whereas the father shows the wild-type sequence at this position. The indel mutation c.1440_1447delinsTAAAAG, leading to original stopcodon lost, results in elongated protein. The patient was found to be heterozygous for this mutation was found in the heterozygous state in the patient (II.1) and the father (I.1). The mother carries the homozygous wild-type allele at this position

Analysis of the *CYP11B1* gene disclosed two novel mutations (Fig. 3): a heterozygous in-frame insertion deletion mutation c.1440_1447delinsTAAAAG in exon 9 inherited from the father and a heterozygous mutation c.1094_1120delTGCGTGCGGCCCTCAAGGAGACCTTGC (p.364_372del) in exon 6 inherited from the mother. These two novel frameshift mutations resulted in both amino acid sequences and splice site changes. Like the deletion mutation inherited from the mother, the mutation c.1440_1447delinsTAAAAG also prolonged the protein sequence. These two novel mutations were not observed in the Exome Aggregation Consortium (ExAC) or 1000 Genomes databases, indicating that the variants were rare. To further determine whether these two mutations were indeed potential pathogenic factors, the *CYP11B1* sequence variations of c.1440_1447delinsTAAAAG and c.1094_1120delTGCGTGCGGCCCTCAAGGAGACCTTGC (p.364_372del) were both automatically predicted by MutationTaster to be disease-causing mutation (Table 2). In addition, we also identified two polymorphic loci in the patient’s *CYP11B1* gene at exon 1 (c.225A > G, p.Leu75Leu) and exon 2 (c.246C > T, p.Asp82Asp).

**Table 2:** Bioinformatics analysis of two novel mutations

Novel mutation	Bioinformatics analysis		
	MutationTaster		Domain Type
	prediction	Score	
c.1094_1120delTGCGTGCGGCCCTCAAGGAGACCTTGC, p.364_372del	disease causing	0.999	K-helix
c.1440_1447delinsTAAAAG, original stop codon lost, results in prolonged protein	disease causing	0.999	C-term

MutationTaster Prediction: polymorphism or disease causing

## Discussion and conclusion

Clinical presentation, laboratory findings, and genetic features indicated the diagnosis of CAH resulting from 11β-OHD in our patient, which appeared to be a compound heterozygote for two novel mutations in the *CYP11B1* gene.

11β-hydroxylase, one of the cytochrome P-450 enzymes, consists of 503 amino acids [12]. To date, a cluster of mutations have been reported, some of which affect the spatial conformation of 11β-hydroxylase to varying degrees, in particular to maintain the conformation of the key region of the enzyme activity [13–17]. Some mutations could change the reading frame, resulting in production of the wrong protein. If the premature termination appears in advance, the protein expression is terminated prematurely, causing the corresponding functional domain of the protein to change or disappear and the enzyme activity to be lost, accordingly [18–21].

In this study, we identified a novel mutation, c.1094_1120delTGCGTGCGGCCCTCAAGGAGACCTTGC in exon 6, which resulted in the deletion of 9 amino acids at position 364_372 (p.364_372del). CYP11B1, a cytochrome P-450 enzyme, uses heme as a prosthetic group to catalyze redox reactions [22]. The three-dimensional structure of the protein shows that I-, K-, and L-helixes contain a highly conserved heme-binding area [22]. The amino acid residues 364_372 constitute the putative K-helix of the CYP11B1 model [22]. The deletion of amino acid residues 364_372 may have resulted in defection of the K-helix. The side chain of R366 faces toward the protein surface, maintaining a positive surface charge. Additionally, the positive surface charge is involved in the CYP-Adx interaction, which is fundamental for CYP11B1 function [14]. A368 was located in a hydrophobic environment and interacted with the hydrophobic side chains of the amino acid residues V336 and L340 located in the J-helix [17]. The change in A368 resulted in a disorientation of the J-helix, I-helix, or K-L loop, containing a meander region and C450 coordinating the heme iron [17]. Changing this structure’s orientation resulted in substantial changes of both the protein conformation and the heme group orientation relative to the enzyme [17]. E371 at the end of the K-helix might be located in the conserved central core of the P450 enzymes, and the protein domain around the conserved central core could be of fundamental importance [15]. We speculate that p.364–372 del could decrease or abolish CYP11B1 activity by preventing the formation of the K-helix, which in turn would affect the tertiary structure of the protein.

In addition, we also identified c.1440–1447 delins TAAAAG in exon 9, leading to the loss of original stop codon and resulting in an elongated protein. L487-A501 is conserved in humans, rats, and mice [22]. One study reported that the last 10 amino acids in the C-terminal region of the *CYP11B1* gene have little effect on *CYP11B1* function [23]. It is not clear whether the C-terminal protein elongation will affect the three-dimensional structure of the enzyme and thus reduce its activity. We speculate that the mutation of c.1440_1447delinsTAAAAG in exon 6 could be the primary cause of the 11β-OHD in our patient. Combining in vitro expression studies with protein structure analysis is a powerful means of providing new insights in the understanding of structural–functional relationships.

The patient in this article took an unnecessarily high dose of dexamethasone over a long period without regular follow-up or dose adjustment, and he ultimately developed iatrogenic Cushing’s syndrome and reduced final height. The treatment of 11β-OHD is generally identical to that of 21-OHD. Glucocorticoid doses should be adjusted to reduce the risk of iatrogenic Cushing’s syndrome and growth impairment, but stress doses of glucocorticoids are necessary in cases of acute illness [24, 25]. Monitoring with plasma DOC and plasma renin activity can be helpful. Additional antihypertensive treatment may be required if blood pressure remains elevated, despite optimal glucocorticoid treatment. Supplemental treatments to maintain the balance of electrolytes and blood pressure include spironolactone, amiloride, and calcium channel blockers [7, 24]. Since the renin-angiotensin system is suppressed in these patients, angiotensin-converting enzyme inhibitors and angiotensin receptor II blockers should be avoided.

The management of CAH can be complicated by iatrogenic Cushing’s syndrome, inadequately treated hyperandrogenism, or both. Patients treated with supraphysiological doses of glucocorticoids not only experienced slowed growth but could also exhibit the signs and symptoms of iatrogenic Cushing’s syndrome. Thus, we emphasized the importance of clinical follow-up. Close clinical monitoring of symptoms and signs, growth and development, and laboratory results are essential to optimize treatment outcomes.

In conclusion, our findings demonstrated the presence of the 11β-OHD phenotype with two novel pathogenic mutations of the *CYP11B1* gene in a Chinese patient. On the basis of our results, the outcome of this study has paved the way for a more efficient diagnosis and genetics counseling for diagnosis of patients with this disorder in China. Further research is required, however, to determine in vitro expression studies and protein structure analysis that may affect 11β-hydroxylase activity.

## Additional files

Additional file 1: **Table S1.** Primers Used for PCR Assay of CYP11B1 Gene. (DOCX 17 kb)

## Abbreviations

11β-OHD: 11β-hydroxylase deficiency
17-OHP: 17α-hydroxyprogesterone
21-OHD: 21-hydroxylase deficiency
ACTH: Adrenocorticotropic hormone
Aldo: Aldosterone
AT-II: Angiotensin-II
BMI: Body mass index
CAH: Congenital adrenal hyperplasia
*CYP11B1*: 11β-hydroxylase gene
*CYP11B2*: Aldosterone synthase gene
DHEA-S: Dehydro-epiandrosterone sulfate
DOC: 11-deoxycorticosterone
E_2_: estradiol
FSH: Follicle stimulating hormone
HDL-C: High-density lipoprotein cholesterol
LDL-C: Low-density lipoprotein cholesterol
LH: Luteinizing hormone
P: Progesterone
PRA: Plasma renin activity
T: Testosterone
TC: Total cholesterol
TG: Triglycerides
UA: Uric acid
