# Supplementary material for: A Chinese patient with 11β-hydroxylase deficiency due to novel compound heterozygous mutation in CYP11B1 gene: a case report
Source: BMC Endocr Disord. 2018 Sep 21;18:68. doi: 10.1186/s12902-018-0295-6 (PMC6151069; doi:10.1186/s12902-018-0295-6)
Supplement: Supplementary file 1 — Table S1. Primers Used for PCR Assay of CYP11B1 Gene. (DOCX 17 kb) [file 12902_2018_295_MOESM1_ESM.docx]

Additional file 1: Table S1. Primers Used for PCR Assay of CYP11B1 Gene.

| Primer | Sense strand | Antisense strand | Fragment size (bp) |
| --- | --- | --- | --- |
| CYP11B1-exon 1-2 | TGACGTGATCCCTCTCGAAG | GGGTCCTGCCTCTCTCGC | 940 |
| CYP11B1-exon 3-5 | AGAAAATCCCTCCCCCCTA | GACACGTGGGCGCCGTGTGA | 1360 |
| CYP11B1-exon 6-7 | GGGGTTTGGATGGGCATTAGGAT | ACCCAGAGAGTAGAGGAACACG | 890 |
| CYP11B1-exon 7-9 | CGACCTGGGCTTCCCATGGATCT | CTGGGACCCTGGGTGCAGAGACG | 1600 |
